# Dealer-customer partnership in rice production demonstration: Assessment of private extension system in Bangladesh

**DOI:** 10.1016/j.jafr.2023.100752

**Published:** 2023-12

**Authors:** Tanjina Parven, Md Safiul Islam Afrad, Shaikh Shamim Hasan, Sajia Sharmin, Muhammad Ashraful Habib, Swati Nayak, Saidul Islam, Aliyu Akilu Barau, Amlan Biswas, Md Shibly Sadik

**Affiliations:** a Department of Agricultural Extension and Rural Development, Bangabandhu Sheikh Mujibur Rahman Agricultural University (BSMRAU), Bangladesh; b Department of Agricultural Economics, BSMRAU, Bangladesh; c International Rice Research Institute, Los Baños, 4031, Laguna, Philippines; d Department of Agricultural Extension and Rural Development, Usmanu Danfodiyo University, Sokoto, Nigeria

**Keywords:** Private extension, Partnership, Dealer, Customer farmer, Rice, Demonstration

## Abstract

Traditional public extension worker-farmer cooperation in rice production demonstration is not working efficiently, therefore, private partnership-based demonstration has been attempted to introduce as its alternative very recently involving dealer-customer farmer. The study evaluated the private extension services rendered through dealer-customer farmer cooperation in Bangladesh. Thirty-three rice seed dealers and ninety-two customer farmers formed the samples for the study. Face-to-face interviews were employed as a quantitative method while focus group discussion was used as a qualitative method in the present study. Involving in the private rice production demonstration approach, customer farmers indicated high profit, greater involvement in decision-making, and improved marketing skills as the major advantages; while the dealers stressed the benefit received by the small farmers, improvement in their decision-making capacity and increased local rice production. However, the slow distribution of inputs during the production period was a weakness in the arrangement, which was mostly because of the dealers' lack of understanding of the customer farmers’ needs. The private extension system being a new concept in the country may be observed over a period and gradually extended to the nooks and crannies of the country.

## Highlights

• Assessment of the private extension system in Bangladesh by analysing dealer-customer partnerships in rice production.
• Rice variety and varietal traits preference of dealers and customer farmers.
• SWOT analysis of private rice production demonstration-based extension system.
• Linkage between the dealers and customer farmers.
• Problems faced in Private Rice Production Demonstration Approach.

## Graphical abstract

**Figure:**
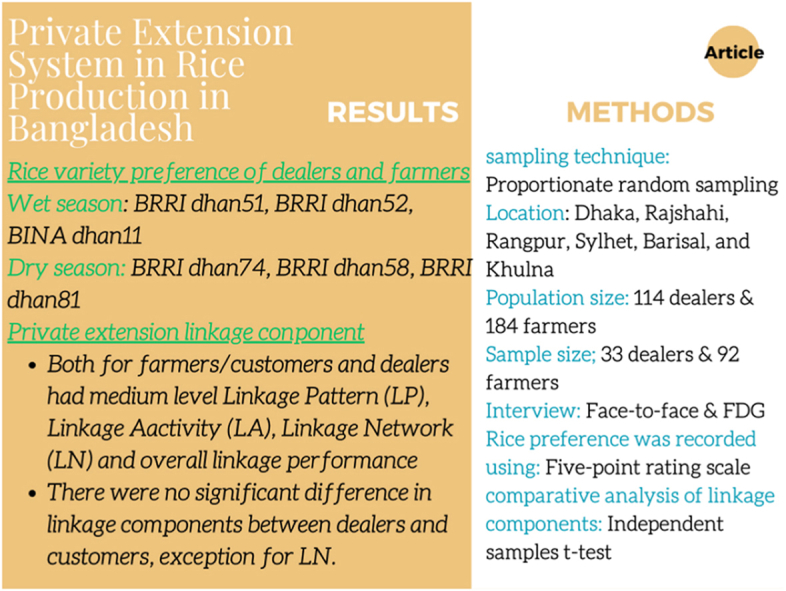

## 1 Introduction

Agriculture in Bangladesh is the main source of food and employment for the rural people, contributing around 11.5% of the country's Gross Domestic Product in the fiscal year 2021–2022 [1]. This output does not satisfy the food and nutrition requirements except for rice production, where the agricultural extension can help maintain a higher production and revenue of this most important cereal crop in Bangladesh through transferring and facilitating information, skills, and technology [2]. As most Bangladeshi farmers do not have much academic education, agricultural extension work becomes the foremost criterion in boosting farmers' productivity [3]. The extension workers have the responsibility to impart required agricultural knowledge to the rural farmers.

Most of the farmers in rural Bangladesh rarely receive agricultural extension services, and the country's agricultural extension services do not function efficiently [4]. Due to the limited availability of public extension workers, farmers or customers seek required information on crop production and protection during the purchase of agricultural inputs from dealers. Furthermore, a greater portion of the farmers don't purchase their agricultural inputs including rice seeds in cash but rather with the assurance of payment after harvest of their product.

National agricultural policies, extension services, and field-level conditions, all have policy action gaps. The rice monoculture occupied 75.0% of the land area, indicating that agriculture has exclusively achieved success in rice monoculture [5] but there has been a gap between the demand and availability of high-quality rice seeds in Bangladesh [6]. Between 2005 and 2012, the ratio of quality seed supply to demand ranged from 25.0 to 58.0% in the Bangladeshi market. Although the public sector's supply of quality rice seeds increased from 24.0 to 49.0% between 2005 and 2012, during the same period, the share of the private sector increased from 1.1 to 12.7%. The private seed distributors took the lost market share of the public seed ventures by the year 2013 [6]. Regardless of origin, government and private agricultural extension services had a substantial negative gap between perceived and expected ratings [4]. However, poor logistic support, lack of fund for actual extension work, poor use of information and communication technology, lack of coordination between research, extension and extension service providers themselves, and political interference were the major difficulties that hampered the service quality of public and private extension service providers, respectively.

Moreover, as agricultural dealers play an important role in crop production, International Rice Research Institute (IRRI) initiated the dealer-customer partnership in Bangladesh in 2019 to (i) identify seed dealers' channels by which they spread information on new technologies and promote the technologies among customer farmers; and (ii) recognize seed dealers’ act as a connecting link between seed users and suppliers. Being the dealer-customer partnership a new concept in Bangladesh, there is a dearth of research on the success or failure of this approach and, therefore, the present study examined the dealer-customer private extension system partnership in rice production demonstration in Bangladesh.

## 2 Methodology

This study focused on areas of Bangladesh where rice is prolific but the production often gets affected by natural disasters. The study was conducted in six divisions of Bangladesh viz. Dhaka, Rajshahi, Rangpur, Sylhet, Barisal, and Khulna. Based on some criteria viz. willingness to participate in the rice demonstration program, high knowledge level on crop production and protection issues and popularity among the farmers, the network partners of IRRI sampled the dealers licensed by the Bangladesh Agricultural Development Ccorporation (BADC). To eliminate the spatiotemporal biases of rice dealers’ responses, data were collected from six distinct divisions during the last four cropping seasons, i.e., Aman 2019, Boro 2019–20, Aman 2020 and Boro 2020–21. A total of one hundred and fourteen dealers were enlisted in the program, among them thirty-three were chosen for the study. Again, ninety-two farmers, the customers of selected dealers were involved with the newly introduced rice varieties demonstration were chosen from the six divisions. Proportionate random sampling technique was followedd in selecting samples from both the groups. Both quantitative data and qualitative information were used in the study. Quantitative data were collected using a semi-structured questionnaire through a face-to-face interview method, while qualitative information was gathered through focus group discussions.

The preferences for rice varieties were measured and ranked based on the mean preference value of individual varieties. Perception of dealers' competencies and positive and negative aspects of the dealer-customer partnership was measured using a five-point rating scale for selected parameters. A five-point rating scale was also used for evaluating the varietal trait preferences of rice by the dealers and their customer farmers. Scores of ‘4’, ‘3’, ‘2’, ‘1’, and ‘0’ were assigned against the five continuums viz., ‘very highly prefer’, ‘highly prefer’, ‘moderately prefer’, ‘low prefer’, and ‘do not prefer at all’, respectively. The trait preference index was measured for each trait by multiplication of their respective weights with the number of responses. Statistics like frequency count, range, mean, and standard deviation was used in the analysis of data wherever applicable. Further, to validate the quantitative results, SWOT (Strength, Weakness, Opportunity, and Threat) analysis was employed through six Focus Group Discussions (FGD) conducted in each division of locale of the study.

To understand the significant differences in the linkage components (activity, network, and pattern) between the two categories of respondents (dealers and customer farmers), a comparative analysis of linkage components was conducted by performing Levene's Test for equality of variances and *t*-test for equality of Means. Levene's test was used to assess the equality of variances within each linkage component for both categories of respondents. Moreover, an independent sample *t*-test was employed to assess the equality of means of linkage component between rice seed dealers and their customer farmers. For both of the tests, the significance level was set at a 5% level. The null hypothesis used in this study was “There is no significant difference in linkage components between dealers and customer farmers”. The severity of problems confronted by each of the respondents was measured by obtaining their opinions on each of the selected problems. The responses to each of the problems were recorded in a five-point rating scale. The extent of responses and corresponding scores were: very severe (4), severe (3), moderately severe (2), low severe (1), and not severe at all (0). The mean score of each problem was calculated to rank the severity of these problems.

## 3 Results

The findings of the study have been discussed in accordance with its objectives.

### 3.1 Customer farmers' and dealers’ choices for rice varieties

**Fig. 1:**
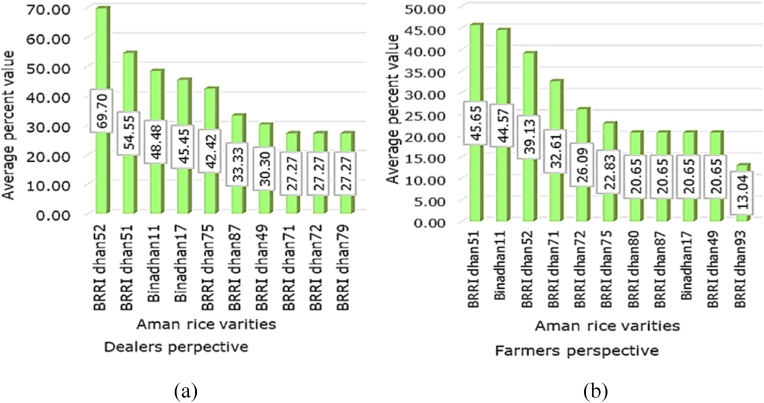
Preferences for different aman rice varieties of (a) dealers, (b) Customer farmers.

Preferences for Aman (wet) rice varieties in Bangladesh were recorded both from customer farmers' and dealers' perspectives. From the dealers’ perspective, BRRI dhan52 was the most preferred rice variety followed by BRRI dhan51 (Fig. 1). BRRI dhan52 was preferred mostly because of its higher yield (5.0 t/ha, after submergence shock) and other characteristics such as clean rice, and medium slender grain shape, translucent, and white grains. According to the customers, BRRI dhan51 was the most preferred variety on account of its high yield (4.0–4.5 t/ha, after submergence shock) and other characteristics such as plant height (90 cm), clean rice, medium bold, and white grains. Another reason was the planting and harvesting time which is from mid-June to mid-July and April, respectively. Long-duration varieties have a higher yield than short-duration varieties. Adaptation to submergence for up to two weeks might be the most dominant reason for the preference for BRRI dhan51 and BRRI dhan52 by the customer farmers.

**Fig. 2:**
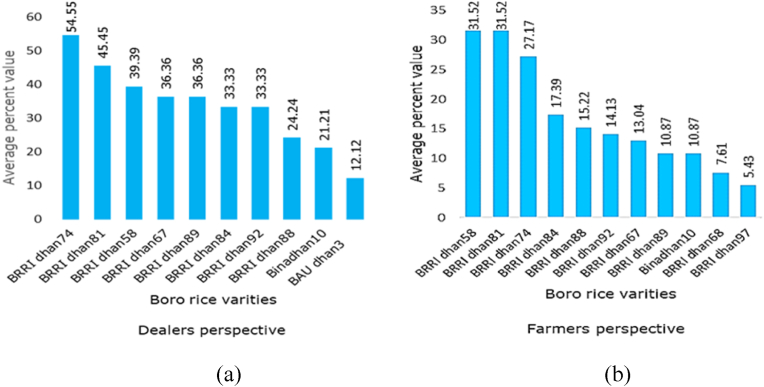
Preferences for different Boro rice varieties of (a) dealers, (b) Customer farmers.

Findings contained in Fig. 2 show that both from dealers' and customer farmers’ perspectives, BRRI dhan58, BRRI dhan74 and BRRI dhan81 were the most preferred Boro rice varieties. BRRI dhan58 is taller than mega variety BRRI dhan29, with grain characteristics similar to BRRI dhan29, but in addition, it has slightly slenderer rice grain, better quality straw and a duration of 150–155 days. The yield of BRRI dhan58 is 7–7.5 t/ha and it is tolerant to medium wilt disease. BRRI dhan74 is stout, and resistant to lodging, with medium broad white rice and 145–147 days in duration. BRRI dhan74 yields 7.1–8.3 t/ha. BRRI dhan74 is Zn-enriched containing Zn at the rate of 22.2 mg per kg of rice. BRRI dhan81 is also stout, with dense tillers, medium slender grains, and 140–145 days growth duration. BRRI dhan81 yields 6.0–6.5 t/ha and is resistant to lodging. These three varieties are planted in mid-November and harvested between mid-April to early May except for BRRI dhan81 which is harvested from May to June. Long-duration varieties yield more than short-duration types. These three varieties are of longer duration and have several desirable traits, hence, very popular.

The role of these high-yielding rice varieties developed by the Bangladesh Rice Research Institute (BRRI) in achieving food self-sufficiency is immense. Also, high-yielding rice varieties developed by the Bangladesh Institute of Nuclear Agriculture (BINA) are being promoted gradually. It takes five to six years for the new varieties to advance and become popular among farmers. Significant improvements have been achieved by IRRI in improving the breeding pathways to impact linking market and farmer preferences to breeding and seed system dissemination [7]. But, in the interest of the country and the farmers as well, the process needs to be expedited. From the analysis, it is evident that customer farmers prefer common and familiar rice varieties. Besides, dealers mostly sell the seeds of popular rice varieties and are not enthusiastic about encouraging farmers to try new varieties. There are several new varieties with favourable traits like high yield and resistance to pests and diseases, but they have not been widely adopted because of a lack of awareness and knowledge about them among farmers.

### 3.2 Varietal trait preference

**Fig. 3:**
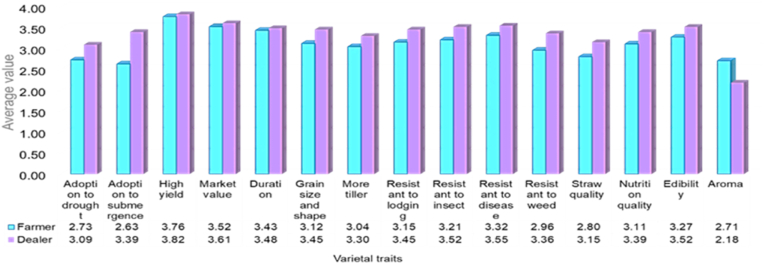
Rank order of the preferences for varietal traits.

The preference for fifteen different traits of rice varieties was recorded through a five-point rating scale (0–4.0) for both customer farmers and dealers. The observed average value of these traits ranged from 2.18 to 3.82 based on which the traits were ranked for both customer farmers and dealers. As shown in Fig. 3, the “high yield” of varieties was found to be the most preferred trait and other preferred varietal traits were “market value”, “resistant to diseases”, “resistant to insects”, “duration”, etc.

Most of the desired attributes were found to be linked with high-yielding attributes of the varieties in the study area. Both dealers and their customer farmers prefer rice varieties for the trait of higher yield and market value but dealers are not as concerned as the customer farmers about the aromatic feature. Otherwise, dealers are more careful about all the specific features of rice varieties than their customer farmers. It might be because the dealers have to learn about the products in detail to provide benefits to the farmers as well as increase their sales.

### 3.3 Strength of private rice production demonstration-based extension system

Information contained in Table 1 show the specific strong/positive points of the private rice production demonstration-based-private extension service system as indicated by dealers and customer farmers.

**Table 1:** Rank order of the strong points of private demonstration-based extension system.

Strength	Dealers		Farmers	
	Average marks	Rank	Average marks	Rank
Accrued more profit from the rice business	3.03	8th	3.14	1st
Increase local rice production	3.33	2nd	2.96	5th
Increase risk-taking ability	3.12	5th	2.89	7th
Improve decision-making ability	3.21	3rd	3.09	2nd
Improve marketing skills	3.18	4th	3.09	2nd
Increase varietal choice and adoption by the farmer	3.03	8th	2.98	4th
Help maintenance of local crop biodiversity	3.06	6th	2.82	8th
Brings benefits to small customers and the local community	3.36	1st	2.90	6th
Increase training facilities for both dealers and customers	2.85	10th	2.39	10th
Increase adoption of new cultivation technique	3.06	6th	2.67	9th

It is evinced from Table 1 that customer farmers considered ‘high profit’, ‘greater improvement in decision-making’ and ‘improved marketing skills’ as the topmost strong points of the private extension system. Improvement in decision-making might be because of customer farmers' informal interaction with their native counterpart seed dealers in different seed production activities viz., quality seed collection, germination, seed bed preparation, seed sowing in the seed bed, maintenance of seed bed, land preparation and seedling tranplanting, irrigation, weed management of rice field, pest management, roguing, proper harvesting, threshing, drying, cleaning and preservation. The marketing skills of customer farmers might be improved due to constant communication with the dealers and their motivational advice and assurances regarding the proper seed packaging, seed distribution, seed marketing time, and actual price determination of rice seeds. On the other hand, dealers indicated ‘benefits to small farmers’, ‘increase in local rice production’ and ‘improvement in decision-making’ as the top three strong points. Small farmers get benefits from this private demonstration system because they are not considered for public demonstration as they belong to poor socioeconomic status. Usually, government extension workers prefer to select large farmers as their demonstration clients which is known wide spread. As most of the farmers in Bangladesh are small and marginal categories and are involved in private demonstration systems, they are encouraged to engage in rice production which ultimately leads to an increase in local rice production. Again, by providing constant advice to the customer farmers, dealers can improve their decision-making capacity in rice seed production, processing, preservation and marketing.

### 3.4 Weakness of private rice production demonstration-based extension service

Both the dealers and the customer farmers observed some specific weak points of the private extension system (Table 2). In Bangladesh, the rice seed production system is complex because of too many actors' involvement in the seed value chain. As the rice seed system is still in its infancy and the private sector is yet to be fully matured, no single actor or sector can currently fulfil the mandated phase-wise seed production and supply cycles. As a result, there exist challenges and gaps in the seed system structure, players’ and institutional engagement, as well as their roles in the efficient operation and supply of high-quality seed varieties.

**Table 2:** Rank order of the weak points of private demonstration-based extension system.

Gaps and issues	Dealers		Customers	
	Average marks	Rask	Average marks	Rask
The slow process of releasing rice production inputs	1.91	4th	1.61	2nd
Weak cooperation of customer farmers	1.94	3rd	1.48	3rd
Poor popularization of newly developed rice varieties	2.36	2nd	1.73	1st
Problems in the transportation of agricultural inputs	1.15	6th	1.14	5th
Negligence of customer farmers	1.42	5th	0.90	6th
Poor agricultural knowledge of customer farmers	2.48	1st	1.16	4th

The dealers considered the ‘poor agricultural knowledge of customer farmers’, ‘poor popularization of newly developed rice varieties’ and ‘weak cooperation of customer farmers' as the major weak points in the private rice seed production demonstration system in Bangladesh. Most of the customer farmers who have been living in rural areas lack formal education and possess poor scientific knowledge of agricultural production, especially rice seed production. The popularization process of newly developed rice varieties is also poor in Bangladesh, therefore, both dealers and customer farmers become supecious in the production, distribution and marketing of choiceable newly released rice varieties. Again, private rice seed demonstration, being a new system for the customer farmers, and the dealers being their native people, they might not cooperate with them with utmost sincerity which is also recognized by the customer farmers. On the other hand, the customer farmers identified ‘delayed supply of rice seed production inputs’ which might be the negligency of dealers of limited availability of the required rice seed production inputs.

### 3.5 Qualitative findings on private demonstration-based extension system

**Fig. 4:**
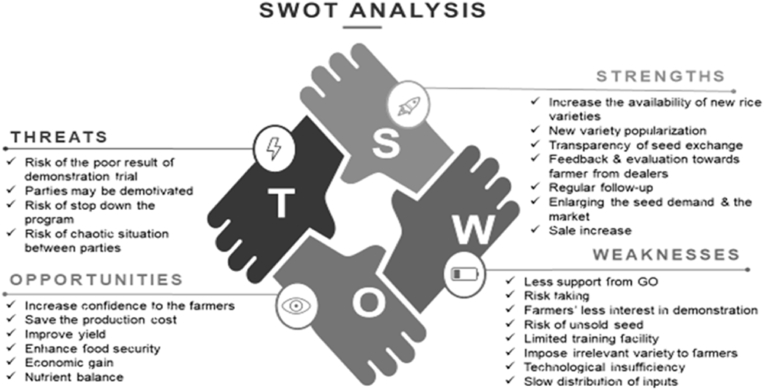
SWOT analysis of the private demonstration-based extension system (based on FGDs) Note: LP= Linkage Pattern, LA= Linkage Activity, LN= Linkage Network.

SWOT (Strength, Weakness, Opportunity and Threat) analysis of rice production demonstration-based private extension service was performed through six focus group discussions (FGDs) at the community level in the six regions of the country. Apart from dealers and customer farmers, representatives from NGOs and reputed persons in the locality were also involved as participants in these sessions. To make it effective, it was ensured that all the participants volunteered for the discussion. The findings of the focus group discussions are presented in Fig. 4.

The strength of the demonstration-based private extension system, as identified by the stakeholders in the system was ‘the availability of new rice varieties’ and ‘their promotion through farm-level trials’. Respective local farmers found within their vicinity that both the customer farmers and dealers worked together and achieved positive results from the demonstration-based private extension system. Therefore, local rice growers become interested in the newly introduced rice variety based on their improved attributes like high yield, good taste and market value. Simple seed exchange process, close follow-up by the dealers, and increase in demand for seeds of new potential varieties are other strengths observed. In this newly introduced demonstration system, dealers just selected the cooperative and innovative farmers based on the previous dealing and provided them with the required amount of newly introduced rice seed for production on a commercial basis. Dealers regularly interacted with the selected seed growers which encouraged them to a great extent. Their simple and cooperative interaction was found attractive by the local rice growers. On the other hand, ‘non-involvement of government agencies’, ‘lack of interest on the part of some customer farmers’, and ‘slow untimely distribution of inputs’ including ‘technological inefficiency dealers’ were observed as major weaknesses requiring improvement. Department of Agricultura extension (DAE), being the relevant public extension agency, with insufficieny of grassroot extension workers (one extension worker for 800–2500 farm families) cann't be involved with the private demonstration system regularly. Participating dealers failed to distribute the essential seed production inputs timely to the customer farmers which became the drawback in performing the activities. Dealers have rarely imparted training on the use of agricultural inputs by their customer farmers. Therefore, they could not solve all the problems encountered by their counterpart.

Few suggestions emerged from the FGD participants towards overcoming the weakness private rice production demonstration system. Multi-location testing of new varieties can be conducted for the functionality of the dealers in terms of productivity and profitability. This would increase the dealers' interest in setting demonstrations with the farmers through newly introduced rice varieties. Promotion and popularization of the new varieties through campaigns at local and regional levels are important because farmers want to purchase only the popular and known varieties and are reluctant to take risks with unknown rice varieties. Older but popular varieties can be improved by the research organizations analysing farmers’ feedback based on their field experience. Farmers are interested only in those varieties that they anticipate are productive and profitable with high market value. Therefore, market demand analysis for seeds prior to the development or improvement of any variety can be given emphasis.

### 3.6 Linkage performance between the dealers and customer farmers

The linkage between seed-producing farmers and private seed dealers was evaluated, which indicates the relationship between dealers and customer farmers. The linkage pattern, activity, and network, as well as total linkage, were evaluated. Linkage patterns included information input, processing, and output. Linkage activity between dealers and customer farmers or were studied on a series of cooperative extension activities including participatory activities, exchange of knowledge and information, and group meetings/discussions. The relationship between input dealers, credit organizations, and non-governmental organizations (NGOs) was studied under the linkage network.

#### 3.6.1 Linkage performance of the dealers

Dealers' linkage performance demonstrated their current position in relation to several linkage components for maintaining interaction with customer farmers. This linkage performance was categorized into three groups (low, medium, and high) based on scores for each linkage component viz., linkage pattern, linkage activity and linkage network.

**Fig. 5:**
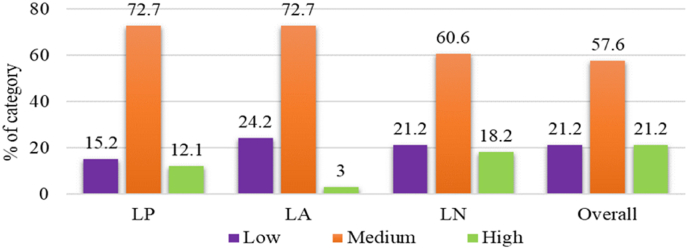
Distribution of the respondent dealers according to their linkage performance Note: LP= Linkage Pattern, LA= Linkage Activity, LN= Linkage Network.

**Linkage pattern:** Dealers' linkage pattern consisted of a combination of information input, information processing, and information output in order to maintain contact with the customer farmers. The dealers’ linkage pattern score varied from 27 to 69, with an average of 48.55 and an SD of 11.848. Based on the scores, the respondent dealers were divided into low, medium, and high. The majority of dealers (72.7%) showed moderate linkage patterns, compared to 15.2% low linkage patterns and 12.1% demonstrated high linkage patterns (Fig. 5). Therefore, most of the respondent dealers had a moderate information input, information processing, and information output pattern while dealing with customer farmers.

**Linkage activity:** The dealers' linkage activity score ranged from 19 to 56, with an average of 39.45 and an SD of 13.59. Based on the scores, the respondent dealers were classified into three categories. The majority of the respondent dealers (72.7%) had medium linkage activity, compared to 24.2% low linkage activity and only 3.0% had high linkage activity (Fig. 5). Thus, respondent dealers maintained a moderate linkage engagement with the customer farmers.

**Linkage network:** Dealers' linkage network included the collaboration with input merchants, credit organizations, and non-governmental organizations (NGOs) for facilitating customer farmers. The dealers' linkage network scores varied from 14 to 70, with an average of 41.48 and an SD of 16.904. Based on the scores, the respondents were also divided into three categories. The majority of the respondent dealers (60.6%) had a medium level of linkage network, compared to 21.2% had a low level of linkage network and 18.2% of them had a high level of linkage network (Fig. 5). So, the majority of dealers had a medium level of interaction with input merchants, credit organizations, and NGOs.

**The linkage performance of the dealers:** The respondent dealers' overall linkage score varied from 69 to 178, with an average of 129.55 and an SD of 36.433. It is evinced from Fig. 5 that the vital part of the respondent dealers (57.6%) had a medium level of overall linkage, compared to each of 21.2% low level and high level overall linkage.

Therefore, the majority of the dealers showed moderate linkage in all three linkage components as well as overall linkage. Only a small percentage of the dealers had excellent linkage performance with their client customer farmers.

#### 3.6.2 Linkage performance of the customer farmers

**Fig. 6:**
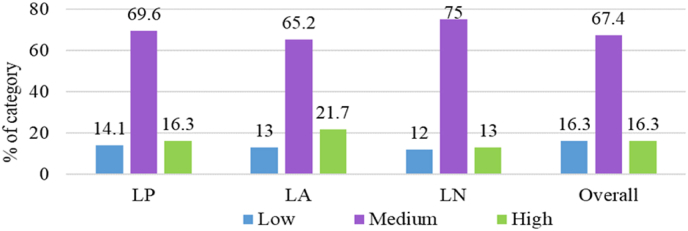
Distribution of the respondent customer farmers according to their linkage performance.

Like-wise the respondent dealers, linkage performance of the customer farmers was also measured by condering their linkage pattern, linkage activity, and linkage network. The linkage performance is the total of all of these four components (Fig. 6).

**Linkage pattern:** The customer farmers' linkage pattern consisted of information input, information processing, and information output for maintaining contact with the dealers. The observed customer farmers’ linkage pattern scores varied from 10.0 to 74.0, with an average of 40.58 and a standard deviation of 13.587. The majority of customer farmers (69.6%) maintained a high level of linkage pattern, whereas 16.3% had medium and 14.1% had a low level of linkage pattern (Fig. 6). Therefore, 85.9% of the customer farmers belonged to moderate to high-level linkage pattern categories.

**Linkage activity:** Customer farmers’ linkage activities included collaborative extension activities performed to maintain a connection with the dealers. The obtained linkage activity scores varied from 4.0 to 56.0, with an average of 29.22 and a standard deviation of 15.78. More than half of the customer farmers (65.2%) were found to be involved with medium linkage activity, compared to 21.7% high and 13.0% low linkage activity (Fig. 6). Therefore, 86.9% of customer farmers had stronger linkage activity with their counterpart dealers. The reason behind this mentionable linkage activity of the customer farmers with their dealers might be the better exchange of knowledge information and joint problem identification.

**Linkage network:** The customer farmers' linkage network consisted of interactions with input dealers, credit organizations, governmental and non-governmental organizations in order to maintain linkage with the dealers. The customer farmers' linkage network scores varied from 5.0 to 80.0, with a mean of 25.60 and a standard deviation of 14.047. Three-fourth (75.0%) of farmer customers had a medium linkage network, compared to 13.0% a high linkage network and 12.0% of them had a low linkage network (Fig. 6). Therefore, more than four-fifths (88.0%) of the respondent customer farmers maintained moderate to high linkage networks with different essential personnel or organizations. It might be possible that this is due to the customer farmers’ potential to maintain relationships with input dealers, credit organizations, and non-governmental organizations in order to build a stronger network for the enhancement of their farming practices.

**The linkage performance of the customer farmers**: The customer farmers’ linkage performance score was calculated by combining all three linkage components. The linkage performance scores of the customer farmers varied from 31.0 to 190.0, with an average of 95.39 and a standard deviation of 38.183. It is evinced from Fig. 6 that the majority (67.4%) of the customer farmers showed moderate linkage performance, while each of 16.3% of them demonstrated high and low linkage performance.

#### 3.6.3 Comparative analysis of linkage components between the dealers and the customers

Comparative analysis of linkage performance of the customer farmers and their counterpart dealers was conducted for investigating the similarities and differences which are also related to the strength and weakness of the newly introduced private extension approach. According to Levene's test, insignificant variance (<0.05) was found in two of the three linkage components viz., activity and pattern but a significant variance (0.19**) in the linkage network was found with the F-value of 5.63 between dealers and farmers. However, an independent sample *t*-test was conducted to compare the means of linkage components for dealers and farmers/customers. In the case of the linkage pattern between dealers and customer-farmers, there were significant mean differences of 8.035, where the p-value (0.003) is smaller than the level of significance. Results indicate that the linkage pattern was very significantly varied between dealers and customer farmers (t-value= 3.0, *p-*value*=* 003). In the case of linkage activity, there were very significant differences between the dealers and customer farmers (t-vale= 3.31 and *p* = .001). The variation was similar in the case of linkage activity. There were very significant differences between the dealers and customer farmers (t-value= 4.83 and *p* = .000).

In the case of linkage activity and linkage network between the respondents, there were significant mean differences of 10.237 and 15.887, respectively with a *p*-value less than the significance level (Table 3). The magnitude of differences in the means for each linkage component was significant. Hence, the null hypothesis could not be accepted. It indicates that the individual data points in each linkage component (activity, pattern and network) have similar levels of dispersion around their respective means but the central tendencies (means) of the linkage components were not the same between the respondent groups.

**Table 3:** The respondents’ comparative analysis of linkage components between the dealers and the customer farmers.

Independent Samples Test										
**Levene's Test for Equality of Variances**				***t*-test for Equality of Means**						
		**F**	**Sig.**	**t**	**Df**	**Sig. (2-tailed)**	**Mean difference**	**Std. Error difference**	**95% Confidence Interval of the difference**	
									**Lower**	**Upper**
Linkage Pattern	Equal variances assumed	.87	.352	3.00	123	.003**	8.035	2.678	2.733	13.34
Linkage Activity	Equal variances assumed	.48	.489	3.31	123	.001**	10.237	3.092	4.116	16.36
Linkage Network	Equal variances assumed	5.63	.019**	4.83	123	.000**	15.887	3.287	9.281	22.49

** Significant Level= 5%.

### 3.7 Problems faced in private rice production demonstration

Both dealers and their counterpart customer farmers encountered different problems. The total score of each problem was summated based on the extent of their respective opinions against each problem statement in a five-point rating scale. The mean value of each problem was calculated. Based on the mean score of each problem statement, the rankings of those problems were done. The notion of this ranking is that the greater the severity of the problem, the higher the mean value of the same.

Results shown in Table 4 indicate that “Cooperation from the DAE and BADC” and “Market price of agricultural produces” were jointly ranked first as the most severe problem as indicated by the dealers. Farmers usually, want to be confirmed that they will obtain benefit in cultivating crops that they will sell at a fair price which sometimes becomes impossible. This occurs because of an uncontrolled marketing system. The second severe problem was “Technical knowledge and information” possessed by the dealers. Agricultural production being scientific in nature requires some technical information to run the production system economically viable, environmentally sustainable and socially compatible. The third problem mentioned by the dealers was the “Training facility”. Training provides functional skills to an incumbent. Training may be formal or informal. Different government and Non-government organizations can enrich the dealers in terms of their knowledge and skill which is very scanty for the dealers. Therefore, they mentioned training as 3rd most severe problem.

**Table 4:** Distribution of the respondent dealers’ problem confrontation in private rice production demonstration.

Sl #	Problem statements	Respondent Dealers	
		Mean values obtained	Rank
i.	Training facility	2.48	3rd
ii.	Technical knowledge and information	2.58	2nd
iii.	Cooperation from customer farmers	1.82	6th
iv.	Cooperation from DAE/BADC	2.70	1st
v.	Monitoring and evaluation of rice production by the customer farmers	1.52	7th
vi.	Transportation facilities	1.39	8th
vii.	Financial capabilities	1.82	5th
vii.	Market price of agricultural produces	2.70	1st
ix.	Customer farmers' interest in adoption of new HYVs	2.42	4th
x.	Business policy	1.76	6th

“Customer farmers’ interest in the adoption of new high-yielding varieties” was also another problem that ranked 4th. A variety of factors may influence their extent of adoption like their age, level of education, farm size, annual family income, cosmopoliteness, contact with the sources of information and organizational participation. Other noticeable problems were “Financial capability” and “Cooperation from farmers”.

It is evinced from Table 5 that “Technical knowledge and information” was the most severe problem encountered by the customer farmers. They also think that dealers are too lacking technical knowledge of newly introduced high-yielding rice varieties. Likewise, the dealers and customer farmers encountered “Training facility” as their second most severe problem. Training makes one capable of doing any specific job in a better way. Both customer farmers and dealers should be imparted with sufficient training on the various aspects of the process starting from the selection of desirable rice seeds to the processing, preservation and marketing of the products at last. Thus, to perform any technical skill, training is inevitable. The 3rd severe problem the customer farmers studied was “Post-harvest management facilities”. It is very crucial that any seeds, especially rice require special pre-harvest as well as post-harvest care. This is because of a variety of pertinent issues like drying, cleaning, grading and, moisture control. If facilities related to these issues are not available to the customer farmers, seed quality will deteriorate loss of germination and other high-yielding features. In the same vein, customer farmers faced some other difficulties like lack of financial solvency, the market price of rice seed followed by additional production care and management for the production of rice seed.

**Table 5:** Distribution of the respondent customer farmers' problem confrontation in private rice production demonstration.

Sl #	Problem statements	Respondent customer farmers	
		Mean values obtained	Rank
i.	Training facility	2.03	2nd
ii.	Technical knowledge and information	2.14	1st
iii.	Additional production care and management	1.50	6th
iv.	Cooperation from dealers	1.40	7th
v.	Monitoring and evaluation of rice production	1.18	9th
vi.	Transportation facilities	0.83	10th
vii.	Financial capacity	1.77	4th
vii.	Market price of rice seed	1.71	5th
ix.	Post-harvest management facilities	1.78	3rd
x.	Sharing rice production knowledge with fellow farmers	1.20	8th

## 4 Discussion

Rice is a staple for the people of Bangladesh and more than 150 million people of the country rely on rice and its supplies account for approximately 70.0% of caloric intake and 58.0% of protein intake [8]. So, rice cultivation and the choice of rice varieties are crucial for farmers. Farmers from all around Bangladesh except the central region preferred BRRI dhan51 and BRRI dhan52 for their special trait of submergence tolerance, as flooding and water logging is not common scenario in the central part [9]. Specifically in southern Bangladesh, BRRI dhan51 and BRRI dhan52 are among the highly adopted rice varieties for the wet season and demonstrated a higher market acceptance rate in the mid-level of the value chain for their medium bold grain and increased consumer demand [10]. Also in Sylhet (northeast), Chapai Nawabganj (west) and Cox's Bazar (south) these rice varieties have a yield advantage, which made them the highly adopted climate-resilient varieties in these regions [11]. Though these varieties take slightly longer to get to maturity farmers often prefer to cultivate them because of their medium bold grain and submergence tolerance capacity of up to 15 days [11].

As new promising boro season rice varieties BRRI dhan58, BRRI dhan74 and BRRI dhan81 were most popular among the dealers and farmers. Varieties such as BRRI dhan72, BRRI dhan80 and BRRI dhan87 were among the most preferred rice varieties in Bangladesh [12]. BRRI dhan58 is taller than mega variety BRRI dhan29, with grain characteristics similar to BRRI dhan29, but in addition, it has slightly slenderer ripe grain, coloured straw and a duration of 150–155 days. The yield of BRRI dhan58 is 7–7.5 t/ha and it is tolerant to medium wilt disease. Although the rate of production of BRRI dhan29 and BRRI dhan58 is higher than other varieties, their traits of a longer growth period, bold grain, and a lack of quality seed are the reasons for the lower adoption rate [10].

Rice variety choice is directed by farmers' preferences for the trait of high yield and early maturity in a variety [13]. Along with yield, varietal traits such as profitability, seed availability and market demand are also found to be the influencing factors of farmers adopting aromatic rice [14]. Farmers in Central Luzon also prefer varieties that give high yield, mature faster (shorter growing season), and have long and slender grains, high milling recovery, and intermediate amylose content [15]. Higher yield and Benefit Cost Ratio are the main driving factors of farmers' preference for HYV rice varieties [16] but farmers also consider tiller quantity, grain size and market value of the rice as important traits [9,16]. Market demand for fine-grain (i.e.,long-and-slender-grain) rice has increased [17] which is a positive sign for farmers who are willing to adopt HYV rice varieties with long slender grain. Even, in the case of sorghum varietal traits such as yield, pest resistance, ability to yield a price premium, and taste were the main varietal attributes factors to the adoption of improved sorghum varieties [18]. Though for variety diffusion and future rice breeding programs, the agronomic, grain quality, and pest and disease resistance traits are paramount, farmers also prefer rice varieties with better taste and straw quality [10]. But farmers grow high-yielding rice varieties, they do not get extra benefits because the price of rice differentiates highly at the retail level and remains much lower for farmers’ raw products [19].

The private sector extension service's strengths include an extensive network of regional employees which help to direct knowledge of what farmers want to increase productivity by the extension workers [20]. In another study, respondent farmers were asked to express their opinion toward private and public agricultural extension in Bangladesh, where the majority of farmers (81.5%) narrated that private sector extension programs are less effective in boosting farmers' farming abilities In contrast, 59.6% of farmers thought the same for public sectors extension [21]. However, in contributing to agriculture in Bangladesh public sector plays a crucial role in extension service delivery compared to private sectors [5] which is a drawback of the private extension services. According to the results of this study, the majority of farmers/customers had a medium performance for several linkage components as well as their overall linkage. One of the most likely explanations is that the farmers/customers had greater networking connections. Therefore, the aforementioned fact reveals that there is potential to upgrade farmers/customers into a stronger linking system by taking them into consideration through systematic extension efforts and motivation to increase their capacities. The connections between different stakeholders have the potential to improve extension organizations' ability to support farm households in achieving their broader livelihood security needs in a more sustainable manner [22]. Moreover, the extension and research agencies in agriculture must function in harmony, which can bring strong linkage for the free flow of information between these extension and research agencies to deliver effective services to farmers [5,20].

The results contained in Table 3 suggested that there was no considerable dispersion within each linkage component between dealers and customer farmers, but the overall central tendencies (means) of the linkage components differ significantly from each other. Extension programs have the daunting task of helping different types of farmers improve their production and their links to the market, despite their vastly different needs and capacities. Farmers often have very little money to invest, which can lead to a long-term dependency on cultivation systems and other service providers [23]. However, a similar study employed an independent *t*-test to find the difference between the four linkage components of the extension agents and farmers, reporting that the t-values for linkage pattern, linkage activity, and linkage network were highly significant, but the *t*-value for linkage technology was not significant [24]. The result has a close conformity to this study, which reported the spread or variability of the data points within each component viz., linkage activity and network was similar or comparable but the average mean values of all linkage components between dealers and customer-farmers are not equal. Another study found that most of the recorded linkage between small-scale producers and value chain actors was positive, where the value was 81.0% positive for output intermediaries, 96.0% for input suppliers (largely cooperatives and agro-dealers) and 100.0% for providers of logistical services [25]. Thus, in all the cases in this study, i.e., very positive and significant differences were observed between the two selected groups. This might be because of the variation in their occupations. The functions they perform in their respective occupations are different. In Bangladesh, most of the farmers, usually produce crops commonly only for their survival but very rarely for commercial purposes. On the other hand, dealers living in rural areas, are generally involved with the selling of agro-inputs and sometimes may be engaged in farming also. Therefore, they maintain their linkage pattern, linkage activity and linkage network according to their own disposal. Farmer networks and seed dealers have frequently used channels for information sharing and seed dissemination. Local seed dealers improved the availability of seed at the community level thus increasing the chance of adoption and spread of improved varieties [26]. Also, linkage networks facilitate information transfer from extension agents to agricultural beneficiaries and farmers play intermediaries in agricultural extension networks [27]. Even to create a hybrid rice risk management framework, the existing Research-Extension-Farmers (REF) linkage, through the Ministry of Agriculture can be utilized but still, it would be adequate as the linkage is very weak at the bottom [28]. However, there are significant flaws and issues in the current seed system, including slow variety development, the release and multiplication of high-quality seeds of farmers' preferred varieties, poor integration of variety development, seed multiplication, and marketing chains, poor popularization of newly developed varieties, a lack of adequate seed storage and processing infrastructure, slow uptake and adoption of varieties, weak enforcement of existing seed policies, and a lack of adequate seed storage and processing infrastructure [29]. Lack of policy in partnership between private and public sectors is a great weakness in the extension service of Bangladesh [5]. However, farmers in the Public-Private Partnership (PPP) project have a better understanding of the benefits of PPP and will utilize the connection for better farm management but the neighboring farmers discouraged the PPP model [30]. It means farmers who do not have any extension contact with the PPP project or their officials are more likely to be discouraged from taking part in a new model. Also, traditional technology and indigenous knowledge of farmers are not accorded with the modern farm requirements due lack of consideration of authorities in the system [20]. Even in crisis moments like post-conflict in Liberia, poor coverage by extension providers was identified as the major weakness in agricultural extension services [31].

Although farmers or customers confront problems in private rice production demonstrations such as getting technical information and training facilities, private extension services have the potential to develop better systems for agricultural agents to run successfully. The most severe problems of the dealers in the rice production business are “lack of cooperation from DAE and BADC, the biggest public rice seed distributor in Bangladesh” and “uncontrolled market price of agricultural produces”. The private rice farming demonstration is new to the dealers, they might not be familiar with pest infestation and their control measures. For proper solution of pest control, they require frequent communication with the aforesaid organizations which they indicated as serious problems. Again, dealers could realize that customer farmers are not interested in agricultural production because of illogical and poor market prices of their agricultural products. However, many of the dealers don't possess this technical knowledge and information through recurrent formal training and informal contact with the grassroot agricultral extension agents. The dealers did not receive sufficient training facilities from responsible organizations, which explains their lack of technical knowledge. Both rice growers and dealers showed improvement in their learning of the subjects designed and the correlation between their knowledge before and after training sessions was highly correlated, whereas dealers expressed their views of moderate use of the training outcomes in determining seed demand, storage, and sale [32]. Even vegetable farmers from the Rajsahi district of Bangladesh also stated their lack of technical knowledge of seed handling [33]. It is very important both for the customer farmers and dealers to have adequate knowledge on private rice production demonstration approach and at the same time knowledge and information on cultivation procedure of the newly introduced high yielding rice varieties. Because demonstration executing farmers have to answer a lot of questions raised by the neighbor farmers. Eventually, customer farmers will try to find the answers from the dealers. Therefore, if dealers are not knowledgeable enough on these issues, the approach will be ridiculous.

## 5 Conclusion and recommendation

Private extension service provision is an emerging trend in the Bangladesh agricultural extension system. Advancing new rice varieties through private extension systems is an emerging need in agricultural sector of Bangladesh. Two major stakeholders in the private extension provision, i.e. the dealers and the customer farmers expressed their opinions on the strength of the private demonstration-based extension system as well as its weaknesses in the present study. It is inferred from the findings that, weak integration of the activities, and supply of inputs, in particular, was one major issue observed. Participation of seed dealers in training, demonstrations and field day profgrams can have potential positive outcomes. Customer farmers may be informed of new varieties by the dealers themselves and also explain to their associated benefits clearly. Once the demand is created, dealers will be motivated to procure seeds of new varieties suitable for their geographies. Such a scenario will lead to higher seed replacement rates and eventually sustainability in production. Private extension services can complement public extension services and help fight against poverty and malnutrition. The dealer-customer farmer partnership is a new concept and can be explored further while addressing the observed weaknesses.

## Declaration of competing interest

The authors declare that they have no known competing financial interests or personal relationships that could have appeared to influence the work reported in this paper.

## Data Availability

Data will be made available on request.
